# Ethical sourcing in the context of health data supply chain management: a value sensitive design approach

**DOI:** 10.1093/jamiaopen/ooaf101

**Published:** 2025-10-21

**Authors:** Camille Nebeker, Jean Christophe Bélisle-Pipon, Benjamin X Collins, Ashley Cordes, Kadija Ferryman, Brian J McInnis, Shannon K McWeeney, Laurie L Novak, Susannah Rose, Joseph M Yracheta, Ishan C Williams, Xiaoqian Jiang, Ellen W Clayton, Bradley A Malin, Bridge2AI Ethical, Legal And Social Implications AI Working Group, Nicholas Greig Evans, Subhashini Chandrasekharan, Ilana Goldberg, Barbara J Evans, Samantha Hurst, Shannon K McWeeney, Aaron Y Lee

**Affiliations:** Herbert Wertheim School of Public Health and Human Longevity Science, University of California San Diego, La Jolla, CA 92093, United States; Faculty of Health Sciences, Simon Fraser University, Burnaby, BC V5A 1S6, Canada; Department of Medicine, Vanderbilt University Medical Center, Nashville, TN 37232, United States; Department of Biomedical Informatics, Vanderbilt University Medical Center, Nashville, TN 37203, United States; Center for Biomedical Ethics and Society, Vanderbilt University Medical Center, Nashville, TN 37203, United States; Indigenous Media Studies in ENVS and Data Science, University of Oregon, Eugene, OR 97403, United States; Berman Institute of Bioethics, Johns Hopkins University, Baltimore, MD 21205, United States; School of Information, University of Texas at Austin, Austin, TX 78712, United States; Knight Cancer Institute, Oregon Health and Sciences University, Portland, OR 97239, United States; Department of Biomedical Informatics, Vanderbilt University Medical Center, Nashville, TN 37203, United States; Center for Biomedical Ethics and Society, Vanderbilt University Medical Center, Nashville, TN 37203, United States; Department of Biomedical Informatics, Vanderbilt University Medical Center, Nashville, TN 37203, United States; Center for Biomedical Ethics and Society, Vanderbilt University Medical Center, Nashville, TN 37203, United States; Homelands of the Oceti Sakowin (Sioux), Native Bio Data Consortium, Eagle Butte, SD 57625, United States; Environmental Health Sciences and Engineering, Bloomberg School of Public Health, Johns Hopkins University, Baltimore, MD 21228, United States; School of Nursing, University of Virginia, Charlottesville, VA 22903, United States; McWilliams School of Biomedical Informatics, University of Texas Health Houston, Houston, TX 77030, United States; Center for Biomedical Ethics and Society, Vanderbilt University Medical Center, Nashville, TN 37203, United States; Law School, Vanderbilt University, Nashville, TN 37203, United States; Department of Biomedical Informatics, Vanderbilt University Medical Center, Nashville, TN 37203, United States; Center for Biomedical Ethics and Society, Vanderbilt University Medical Center, Nashville, TN 37203, United States; Department of Computer Science, Vanderbilt University, Nashville, TN, 37212, United States

**Keywords:** artificial intelligence, machine learning, health data repository, ethically sourced, research ethics

## Abstract

**Objective:**

The Bridge2AI program is establishing rules of practice for creating ethically sourced health data repositories to support the effective use of ML/AI in biomedical and behavioral research. Given the initially undefined nature of ethically sourced data, this work concurrently developed definitions and guidelines alongside repository creation, grounded in a practical, operational framework.

**Materials and Methods:**

A Value Sensitive Design (VSD) approach was used to explore ethical tensions across stages of health data repository development. The conceptual investigation drew from supply chain management (SCM) processes to (1) identify actors who would interact with or be affected by the data repository use and outcomes; (2) determine what values to consider (ie, traceability accountability, security); and (3) analyze and document value trade-offs (ie, balancing risks of harm to improvements in healthcare). This SCM framework provides operational guidance for managing complex, multi-source data flows with embedded bias mitigation strategies.

**Results:**

This conceptual investigation identified the actors, values, and tensions that influence ethical sourcing when creating a health data repository. The SCM steps provide a scaffolding to support ethical sourcing across the pre-model stages of health data repository development. Ethical sourcing includes documenting data provenance, articulating expectations for experts, and practices for ensuring data privacy, equity, and public benefit. Challenges include risks of ethics washing and highlight the need for transparent, value-driven practices.

**Discussion:**

Integrating VSD with SCM frameworks enables operationalization of ethical values, improving data integrity, mitigating biases, and enhancing trust. This approach highlights how foundational decisions influence repository quality and AI/ML system usability, addressing provenance, traceability, redundancy, and risk management central to ethical data sourcing.

**Conclusion:**

To create authentic, impactful health data repositories that serve public health goals, organizations must prioritize transparency, accountability, and operational frameworks like SCM that comprehensively address the complexities and risks inherent in data stewardship.

## Background and significance

Biomedical research has undergone significant transformations over the past few decades, driven largely by advances in technology and aspirations toward more precise and personalized medicine.^1^ Modern technologies, including machine learning (ML) and generative artificial intelligence (AI), most recently those based on large language models, are important components of this data-driven landscape. These changes are propelled by newly accessible sources of health data, including electronic health and genomic records, digital health systems (eg, mobile applications, pervasive sensors), publicly accessible environmental data, and insurance claims. The potential benefit of this transformation includes improved detection and diagnosis (ie, medical images to diagnose diabetic retinopathy,^2^ personalized treatment (eg, genetic analysis to identify the best cancer treatment for the patient),^3^ and predictive analytics (ie, ability to accurately predict sepsis among patients).^4^ However, if appropriate care is not taken, potential pitfalls are possible, such as the deployment of algorithms that do not work equally well for all groups of people.^5^ For example, researchers showed that a widely used algorithm that underestimated the health needs of African American patients led to unequal allocation of healthcare resources.^6^

## Objectives

In this paper, we propose how risks of algorithmic bias and other downstream harms can be reduced by applying learnings from the supply chain management (SCM) literature. In response to the National Institutes of Health’s adoption of the term “ethically sourced” with respect to creating 4 health data repositories, we sought to define this term through a conceptual investigation. SCM is where the term ethical sourcing has its roots and has recently been used to frame ethical data science practices, albeit differently from our work.^7^ Applied to the development of a health data repository, the SCM process would involve evaluating the demand for a health data repository and then, if warranted, determining the appropriate data sources followed by data acquisition and collection for representativeness; preprocessing data to assess bias or disparities; validating data via quality checks for accuracy and completeness; and steps to develop and test an algorithm on different patient populations to assess performance across demographics. Drawing from SCM literature, we describe principles and steps to guide the ethical design and management of health data repositories intended for use in AI/ML model training. While not a perfect analogy, there are powerful lessons to be learned from practices that contribute to ethical sourcing in SCM that could inform the debate about creating and using a health data repository for AI/ML. The processes described in this paper are specific to the pre-modeling phase and do not address downstream ethical, legal, and social implications (ELSI) associated with AI/ML model development or the creation and/or use of derivative datasets.

### National Institutes of Health’s Bridge2AI

In 2022, concurrent with the growing production of data for AI/ML, the National Institutes of Health’s (NIH) Bridge2AI program was established to develop standards for ethical, inclusive, and equitable practices to guide the design and creation of health data repositories planned for future AI/ML model development. The challenges of creating effective and usable health datasets through a process that adheres to ethical standards for ML/AI use are numerous, while our current regulatory and logistical structures are insufficient. By defining and initiating practices to address these new challenges, Bridge2AI will establish new standards for responsible data stewardship, safeguarding the integrity and ethical use of its data repositories and fostering a more transparent and collaborative approach to healthcare innovation. Concurrent with developing new standards, the Bridge2AI program is supporting the development of 4 data repositories. These are referred to as grand challenges (GCs), with each taking a unique approach to acquiring and managing data for use in AI/ML training and model development. The datasets include human cell lines to study functional genomics, (Cell Maps for AI [CM4AI]) and de-identified patient data from intensive care unit (ICU) records (Collaborative Hospital Repository Uniting Standards [CHoRUS]). Two GCs are recruiting participants to contribute to datasets that include relatively new data types for use in research including retinal images (Salutogenesis—Artificial Intelligence Ready and Equitable Atlas for Diabetes Insights [AI READI]),^8^ voice recordings (Precision Public Health—Voice as a Biomarker of Health [B2AI-Voice]), as well as traditional clinical and sociodemographic data.

An overarching objective of the Bridge2AI program is to “establish rules of practice to guide inclusive, trustworthy, and successful use of AI across biomedicine and behavioral research.”^9^ Of its 5 goals, one is to generate “ethically sourced, trustworthy, well-defined and accessible”^9^ datasets for the development of artificial intelligence models and the training of machine learning tools. However, the term “ethically sourced” was not defined at the program’s onset. The work to define and characterize “ethically sourced” has occurred concurrent with the work to design and build the 4 health data repositories. While this opportunity to develop recommendations based on the real-world challenges and experiences of these projects allows for practical and useful recommendations for future health data repository initiatives, it also posed challenges in implementation.^10^

This paper applies Value Sensitive Design (VSD),^11^ which is an approach to technology design that systematically accounts for human values throughout the design process. We focus narrowly on the conceptual stage of identifying stakeholders and values where the identification and integration of SCM values and principles inform considerations for the ethical sourcing, processing, and governance of a health data repository. By aligning the logistics and traceability aspects of SCM with values traditionally characterized in health research such as privacy, equity, and accountability, we introduce a novel framework to address critical ethical challenges in health data stewardship. This conceptual investigation seeks to illuminate the socio-technical and ethical complexities of designing repositories that respect the rights of individuals while ensuring data integrity and accessibility for research and public health purposes.

## Methods

This conceptual investigation applied a VSD approach to explore the intersection of ethical health data repository management and SCM. The process involved identifying those who would be affected by the development of a health data repository for ML training with a goal of surfacing values and tensions. We initiated discussions among an interdisciplinary group associated with the Bridge2AI consortium (ie, ethicists, clinicians, social scientists, engineers, privacy experts, and data scientists), many of whom work with diverse consumer, patient, and community groups. These conversations prompted discussion of the term “ethically sourced,” which led to exploring the SCM literature for values and operational principles relevant to health data repositories. These values, including traceability, quality control, and risk mitigation, were assessed for their applicability in the context of health data sourcing and governance.

Creating a value-sensitive framework involved mapping ethical principles of biomedical research to the stages SCM (see Table 1). Potential value tensions, such as the tradeoffs between making data publicly available and setting up appropriate access pathways considerate of data contributor privacy were explored. A use case was created to test the applicability of the framework and refine its components. Finally, we synthesized these insights into actionable guidelines for designing ethically sourced health data repositories, ensuring that the resulting framework addresses both operational and ethical dimensions comprehensively.

**Table 1.** Mapping SCM steps, values, human resources, and expertise to health data repository development.

SCM step	Activities in SCM	Health data repository counterpart	Ethical sourcing values	Actors	Required expertise
Demand planning	Forecasting demand, assessing market needs, planning production and inventory levels, identifying health data needs, understanding research objectives	Identifying need for certain health data, planning data collection strategies, engaging with healthcare providers	Transparency, traceability, equity, community involvement	Clinicians, researchers, data scientists, sustainability experts, business analysts, public	Data science, biomedical informatics, ethics, law, and risk management
Supply chain design	Designing supply chain network, selecting suppliers and logistics providers, designing data architecture, documentation processes and infrastructure, establish governance	Designing data collection, storage, and processing networks, respecting sustainable practices	Effective data collection, storage, and processing networks, respecting values that lead to sustainable practices	Data architects, IT experts, public input	Ethics, law, network optimization, data management, risk management
Sourcing	Procuring raw materials, negotiating contracts, ensuring supplier compliance with ethical standards, acquiring health data from various sources	Acquiring health data from diverse sources, ensuring data quality and integrity, adhering to ethical data practices	Diverse sources, data quality, integrity, confidentiality, privacy	Data sourcing managers, data governance teams, regulatory bodies	Regulatory compliance, ethics, law, procurement, data quality assurance
Manufacturing	Transforming raw materials into finished goods, monitoring production processes, implementing quality assurance measures, processing and transforming raw data into usable formats	Processing and transforming data into usable formats, ensuring data quality and security during transformation	Data quality, security, transformation	Data engineers, data scientists	Data processing, data transformation
Quality control	Inspecting products for defects, ensuring compliance with standards, conducting testing and certification, validating data quality and integrity	Ensuring data quality, accuracy, and compliance with regulations, following standards for data quality and regulatory compliance	Data quality, accuracy, compliance, standards	Data quality managers, auditors	Data validation, data certification
Distribution	Determine access requirements (ie, open, controlled, restricted), establish licensing requirements, implement data use committee review processes, verify data user competency	Securely and timely distributing data to ML/AI model developers, considering environmental sustainability and social responsibility	Secure data distribution, environmental sustainability, social responsibility	Data distribution managers, IT teams	Data transfer, data security
Inventory management	Monitoring data usage and updates, effectively managing data inventory	Maintaining data accuracy and visibility, applying ethical data management principles	Data waste minimization, data accuracy, visibility	Data inventory managers, governance team	Data inventory management, data analytics

## Results

### Origin of ethical sourcing in SCM

The term “ethical sourcing” arose in the 1990s, but its origins can be traced back to the 1960s and 1970s due to concerns about labor rights, environmental protection, and social responsibility.^12^ During this time, the concept of corporate social responsibility began to take shape.^13^ In the 1980s, there was an increased focus on socially responsible investing, and the practice of “ethical sourcing” was part of the commitment companies made to address concerns about labor rights and practices as well as environmental degradation and sustainability.^14^ These actions were in response to the exploitation of child labor in sweatshop conditions by manufacturing companies.^15^ To address these concerns in SCM, principled frameworks have been advanced to help companies adopt ethical practices,^16^^,^^17^ technologies, including blockchain, have been designed to increase transparency,^18^ among other improvements to promote responsible business practices.^19^ In practice, these steps are not perfect and merely labeling products as “ethically sourced” can create a facade of prioritizing human values over profit, akin to “greenwashing.”^20^ We suggest that “ethics washing” constitutes a newer form of deceit taken by data industries in their efforts to appear trustworthy.^14^^,^^21–23^

### Ethical sourcing in digital/AI health research

Rhetoric around “ethical sourcing” occurred in response to forms of exploitation in business. In health data SCM, exploitation can take different forms. The following are examples:

- Unauthorized use of an individual’s health data. Some argue that data exploitation may involve the unauthorized or unfair use of an individual’s health data that occurs without consent or other means of demonstrating respect.^24^
- Creation of datasets that do not contain sufficient information needed to apply to all people to whom the AI would be used. Data bias, which can occur when certain groups are mis- or under-represented in datasets, is another form of potential harm in the biomedical context. Systemic bias in the collection and management (inclusive of storage and sharing) of data has led to unequal treatment and outcomes for certain groups like women, minorities, and low-resourced populations.^25^
- Producing inaccurate, incomplete, or misleading data collections. A parallel with the exploitation of the environment in the business domain in biomedical data is “data pollution” where inefficient or uncoordinated data collection, storage, and analytic processes lead to putting inaccurate, incomplete, or misleading biomedical data into the supply chain, which can contaminate downstream applications.^26^
- Not properly protecting data processes from cybersecurity threats. Cybersecurity threats are another example as these threats may lead to a health data breach from not protecting sensitive health data from unauthorized access, theft, or manipulation.^27^ Indeed, the issue of cybersecurity is compounded when the new data life cycle is considered.

These examples highlight the importance of a data lifecycle in the ethical sourcing of health data, from demonstrating respect to people at the outset to monitoring for possible biases, inaccuracies, and cybersecurity threats, among other concerns. In industry, a product is generally consumed, degraded, or minimally recycled and thus has a finite lifecycle. We argue that a value sensitive approach can be taken to prioritize research ethics across the health data supply chain.^27^

### SCM guiding principles and core values

Values emerging from the SCM history and the evolution of ethical sourcing translate to practices that allow for and support documentation of open and honest communications across all interactions.^28^^,^^29^ A primary objective of SCM is to know the business demand and subsequent activities beginning with procurement, identification of materials, manufacturing of products through to distribution and retail outlets. The goal is to deliver a defect-free product to the customer faster and more reliably than the competition. The value of *accountability* involves assessing and managing risks and taking responsibility for actions and decisions and being answerable to consumers for the impact of those actions.^30^^,^^31^ To be *sustainable* involves managing supply chains in a way that minimizes environmental harm and promotes social responsibility.^32^^,^^33^  *Integrity* requires that those involved are honest, demonstrate a commitment to purpose over profit and demonstrate rigor in management processes, including avoiding corruption and bribery.^16^  *Respect for human rights* acknowledges that supply chains should not engage in human rights abuses, including forced labor, child labor, and human trafficking.^34^ By integrating ethical sourcing practices into the supply chain operations, companies are better able to identify and mitigate risks, improve brand reputation, and contribute to a more sustainable and equitable global economy.

The SCM values and related practices also align with accepted ethical principles in biomedical research, which provide scaffolding for others creating “AI-ready” datasets.^35^ When appropriate, ethical practices may include seeking perspectives of indigenous and other communities who are under-represented in biomedical research. This may include exploring perspectives of authority, collective benefit, self-determination, and intergenerational well-being. By including perspectives of diverse groups of constituents during the planning phase, complementary as well as competing goals can be identified. A robust governance process will anticipate these tensions and have strategies in place to mitigate and manage conflicts among those involved across the pre-model phases.

### SCM process

The first step in SCM is *demand planning*. During this phase, forecasting demand, assessing and mitigating downstream risks, planning production, and inventory management are mapped out.^36^ Next is the *supply chain design* during which time the trusted suppliers, manufacturers, and logistics providers are identified, and contracts are initiated^37^ with clear plans for product safety requirements, distribution, and timelines. *Sourcing* is the process of selecting or acquiring the raw materials, components, or finished goods from the suppliers, which are then transformed from raw materials to the finished product during the *manufacturing* step.^38^ Once the product is manufactured, *quality control* is conducted to determine that quality standards are met. This phase can involve inspection, testing, and certification processes.^39^ Once the product quality is established and expectations aligned, the *distribution* process is initiated. During distribution, the product is transported, stored and *inventory management* begins.^39^

We suggest that the SCM process provides useful insights and the foundational framework components that are applicable to health data repository ethical and responsible design and build activities. Learning from other sectors with a history of developing practices that align with ethical sourcing, even when the context is different, may inform a structured pathway for building health data repositories for use in AI/ML. The following use case depicts a prospective data collection context where participants are enrolled for the purpose of contributing personal health data to a repository that will be used for machine learning training purposes to improve human health—similar to 2 Bridge2AI grand challenges.

### Use case: health data repository development

Steps used in SCM applied to health data repository development:

- **Demand Planning**: Identify the need for a health data repository, assess and mitigate downstream risks of harm, assess the target user population, and forecast the demand for data and analytics and specific use-cases. This step includes planning for the data repository prioritizing patient/participant respect, privacy, security, and autonomy. Evaluating potential risks and benefits of data collection and sharing may include consulting with patients and other constituents in the decision-making process.
- **Supply Chain Design**: The data repository architecture must be explicitly designed to operationalize transparency, accountability, and fairness, embedding bias identification and mitigation strategies throughout data collection and processing to ensure equitable and trustworthy outcomes.
- **Sourcing**: Identify and procure data from various sources, such as human participants via survey, sample collection, and pervasive sensors, as well as existing sources ie, electronic health records and claims data. Ensure informed consent is obtained from human participants, when appropriate, and that data are collected and stored in a way that respects participant agency and privacy. Consider the potential power dynamics between data collectors and participants.
- **Data Processing**: Clean, transform, and integrate data from various sources into a standardized format. Make sure that data processing is done in a way that minimizes bias and respects the diversity of the data sources. Plan for potential risks of data aggregation and de-identification and develop mitigation strategies that are aligned with and support patient safety and wellbeing.
- **Quality Control**: Validate data quality, identify data bias, ensure data security and privacy, and perform data governance. Develop quality control measures that prioritize patient safety and well-being. Consider the potential consequences of data errors or biases and implement measures to mitigate them.
- **Distribution**: Implement robust governance frameworks that control and monitor data access rigorously, ensuring accountability and preventing unauthorized use or misuse of sensitive health data. Provide access to data and analytics to authorized users, such as researchers, clinicians, and policymakers. Develop practices for data access to be controlled and monitored such that unauthorized use or misuse is prevented. Consider the potential risks of data sharing and implement measures to mitigate them.
- **Inventory Management**: Manage data inventory levels to ensure timely and adequate supply of data and analytics. Adopt data inventory management practices that enhance transparency, enforce accountability, and promote fairness, thereby ensuring equitable availability and preventing data scarcity or overload that could bias research outcomes. Consider the potential consequences of data scarcity or abundance and implement measures to mitigate them.

This use case illustrates the intricate, multi-step operational processes inherent in SCM, demonstrating its power as a structured framework for managing complex health data ecosystems. Both demand planning and forecasting determine value and anticipate the supply demands. Assessing the need to acquire raw materials or personal health data is necessary as is quality control and testing to facilitate the products being safe, effective, and of acceptable quality. For the health data repository, the data include digital records of individual-level human data, as such, the regulatory environment is important to consider. This includes regulations for protection of human research participants (45 CFR 46), the Health Information Portability and Accessibility Act of 1996 and, in many cases, state privacy protection regulations, which may be more conservative than federal requirements. The last step in the SCM is making the product available. In the case of a health data repository, there will be pathways for access by the data users.

This case begins to depict what ethical sourcing looks like, even though the nature and scope differ from a business case of manufacturing a consumable product. Beyond analogy, SCM provides a practical, operational framework whose values and processes directly inform the ethical design and management of health data repositories. Although SCM employs different materials and terminology, health AI practitioners can directly apply its proven strategies and operational lessons to achieve authentic ethical sourcing and robust bias mitigation in health data supply chains. In addition to SCM steps, it is important to acknowledge the specialized expertise needed, including specific knowledge and skills needed to carry out each step in the process. Building from the example above, Table 1 depicts the process and how it applies to the health data chain management considering the values, people and skills needed to accomplish the activities associated with the repository development.

### Influences of ethical sourcing by actors involved across stages of repository development

Creating an ethically sourced health data repository requires coordinated engagement of diverse actors-including data contributors, regulatory bodies, ethicists, dataset creators, data owners, stewards, curators, users, the public, and sponsors—each playing critical, interdependent roles throughout the data lifecycle. The complexities require the involvement of human resources who have unique roles and responsibilities across the acquisition, management, and eventual use of health data repository. Given this complexity, it becomes increasingly important to ask what ethical norms and legal rules should guide the processes involved across the lifecycle. By recognizing the limitations and potential influences of each group, the health data repository governance can be designed to identify and mitigate potential biases and serve the greater good. Tables 2-4 describe the actors involved across each stage of a repository design and build process. Included is a description of their roles, challenges faced and strategies to mitigate these challenges. Each table includes an open question to consider that may influence ethical sourcing across the planning, collection and pre-use stages of the health data SCM.

**Table 2.** Planning actors stage.

Actor	Role	Challenges	Mitigation strategies	Values to draw on when conflicts arise	Open question
Sponsors	Provide financial and logistical support for health data repositories.	Conflicts between quick data access and responsible management.	Allocate resources for planning and infrastructure to support ethical practices.	Transparency (as a procedural value): sponsors should be open about why they favor quick data access. Should assess if privacy, confidentiality are at risk	What funding models ensure ethical principles will guide repository planning and implementation?
Data creators/collectors/designers/users	Collect, generate, or acquire data that provides value to users.	Limited expertise, biases in data collection, and conflicting vendor agreements.	Balance innovation with ethics using a team-science approach.	Effectiveness (as a procedural value) but not at expense of respect for persons (data donors)	How can innovation proceed without compromising trust and mitigating potential harms?
Community representatives	Represent contributor and community interests in governance.	Community advisory board composition and representation concerns.	Enhance informed consent and respectful data management.	Community involvement and equity. Community involvement should facilitate considerations of equity	What practices best demonstrate respect for contributors and their communities in repository creation and use?
Regulatory and ethics review boards	Ensure ethical and regulatory alignment of study protocols.	Regulatory limits on considering long-term societal impacts.	Build capacity for ethical reflection across stakeholders.	Transparency, consent, privacy, and respect for persons (data donors)	How can academic medical institutions support capacity building for ethical practices throughout the health AI lifecycle?

The planning stage sets the foundation for the health data supply chain. Four key actors introduce tensions that must be mitigated to ensure ethical data sourcing.

**Table 3.** Data collection actors stage.

Actor	Role	Challenges	Values to draw on when conflicts arise	Mitigation strategies	Open questions
Data contributors	Provide personal health data to the repository through informed consent or data sharing.	Ensuring data literacy, technology literacy, and research literacy for voluntary participation. Broad consent lacks specificity to research questions. Delivery formats affect the informed consent process. Maintaining respect for contributors despite limited consent processes.	Integrity, Responsibility, Responsiveness	Educate contributors on data literacy. Develop hybrid consent processes combining automated and human support. Implement ongoing communication with contributors to inform them about data usage.	What are the data collector’s responsibilities to acquire, manage, and allow access to data given the limitations of informed consent?
Data curators	Organize, annotate, and maintain collected data.	Ensuring curators have adequate domain expertise, resources, and infrastructure to maintain data quality. Ethical concerns in student training scenarios where responsibilities might be overlooked.	Transparency, respect for persons, privacy, equity	Implement certification programs to ensure ethical data curation. Develop protocols for training and resource allocation to support curators’ roles.	What skills and training are needed by those who curate data, and what protocols will support their role?

Data contributors and curators play an important role in the ethical collection and curation of the health data repository.

**Table 4.** Pre-use actors stage.

Actor	Role	Challenges	Values to draw on when conflicts arise	Mitigation strategies	Open questions
Data stewards	Oversee data management and compliance with policies and regulations. Enforce governance and communicate responsibilities across the data supply chain.	Requiring technical expertise, governance authority, and consistent communication across the supply chain.	Transparency respect for data donor, privacy, confidentiality. Data stewards should be transparent about governance processes which should respect donors.	Provide technical training, clear governance protocols, and well-defined responsibilities for all stakeholders.	Are licenses or data use agreements sufficient to convey responsibility when the public becomes data stewards?
Data access committees	Ensure consent, deidentification, and transparency in data access decisions. Manage institutional memory and balance social good, health research, and licensing agreements.	Balancing ethical access criteria with advancing research and avoiding stigmatization. Accountability for institutional memory and licensing agreements.	Accountability In addition to transparency, respect for data donors, privacy, and confidentiality, data access committees should value accountability and be accountable.	Develop access criteria that prioritize ethics and employ licensing agreements with accountability measures.	What are the risks and benefits of granting access to sensitive health data to for-profit organizations vs academic or non-profit institutions? What procedures can effectively restrict or revoke access?
Data users/consumers	Use data for analysis and decision-making while promoting transparency, fairness, and explainability in models. Address potential biases and disparities in healthcare outcomes.	Ensuring transparency, explainability, and fairness in machine learning models while mitigating biases and disparities.	Transparency, explainability, fairness	Adopt mechanisms to ensure model fairness, transparency, and regular evaluations to address biases.	How can data users guarantee transparency, explainability, and fairness in machine learning models, especially regarding healthcare biases?

During the pre-use stage, data stewards, data access committees, and data users are influential in shaping ethical data access and use.

## Discussion

Ethical sourcing is integral to every stage of health data repository development, beginning with demand planning, followed by the establishment of protocols for appropriate sourcing (acquisition, collection) and data management (pre-processing, curation, documentation, stewardship, access). These steps precede model development and form the foundation for traceability, transparency, and robust data documentation. Ethical sourcing also involves identifying the roles, responsibilities, and qualifications of individuals at each stage, from planning to data use. Conceptually, this approach aligns with the aspirations of the Bridge2AI program by contributing to trustworthy and accessible AI. Addressing potential biases during pre-model stages enhances data reliability and ultimately improves model outcomes.

Acknowledging the challenges of SCM is crucial, particularly the risk of “ethics washing” and the complexities inherent in health data management.^13^ Prioritizing transparency and aligning organizational values throughout the data repository development process can foster perceptions of authenticity and trustworthiness. To support ethical data sourcing, capacity building should be emphasized across all stages, promoting responsible and sustainable practices. Furthermore, organizations must remain committed to enhancing visibility and accountability throughout the health data repository development process.

Applying the SCM approach to health data repository creation, in anticipation of AI/ML development, establishes an operational framework that systematically supports responsible data acquisition and management in the pre-model phase. Key SCM components, such as data provenance traceability and continuous risk assessment, are facilitated by practical tools like datasheets^40^ and healthsheets,^41^ which bolster transparency and accountability. SCM’s focus on traceability aligns with VSD, emphasizing the importance of transparency. Skills vital for SCM—market assessment, data procurement, partner engagement, and continuous improvement—are also crucial for developing and managing health data repositories. Nevertheless, challenges persist in incorporating SCM into health data chain management. A significant concern is “ethics washing”—the superficial adoption of ethical principles without substantive changes.^13^ To ensure ethically sourced data, repositories should align acquisition practices with both ethical and legal standards, aiming to advance healthcare knowledge and practice.

Leveraging VSD, we propose explicit mappings of core values to SCM stages to help surface and address conflicts or competing priorities during repository development. Actively engaging diverse stakeholders, including patients, healthcare providers, and researchers, ensures that their values and perspectives are integrated into governance structures. An ethically sourced health data repository documents data provenance, intended uses, and, when appropriate, involves stakeholders who will benefit from the derived knowledge. Ethically managed data must facilitate use while protecting contributor privacy (through curation, secure documentation, and storage) and avoiding harm or stigma to communities. Building a trustworthy repository requires transparent, accountable relationships among contributors, stewards, managers, and end users—fostering mutual trust essential for responsible AI/ML development.^42^ These relationships must be built on trust, respect, and a commitment to transparency and accountability to the people who are going to use and be affected by these tools.^43^^,^^44^

Despite challenges, SCM’s application to health data repository design offers transformative potential for embedding ethics, improving data quality, and enhancing health outcomes. By prioritizing ethical practices, organizations can build trust, improve data quality, and ultimately, improve health outcomes.^45^ Moreover, the use of SCM can help to identify and mitigate issues related to data bias and discrimination, which are critical concerns in the development and use of ML/AI in healthcare.^46^ By proactively addressing SCM’s challenges and limitations, organizations can cultivate more authentic, effective, and resilient health data management practices.

### Limitations

The processes described in this paper are specific to the pre-model data phase and do not address downstream ethical, legal, and social implications associated with model development or derivative dataset creation and use, which can be important to patients and clinicians.

## Conclusions

This paper demonstrates the value of bringing theories, frameworks, and insights from other disciplines into medical informatics. SCM provides a practical, operational framework for systematically addressing ethical challenges in health data repository development. By applying SCM’s end-to-end principles, such as traceability, transparency, and risk mitigation, stakeholders can embed ethical sourcing practices into every phase of pre-model data stewardship, from demand planning to distribution. These steps directly parallel modern SCM’s digital transformation, where tools like datasheets, healthsheets, and real-time analytics ensure accountability and provenance tracking, mirroring healthcare’s need for auditable, bias-aware data ecosystems.

## Data Availability

The data underlying this article are available in the article.
